# Supplementary material for: Wheat Developmental Traits as Affected by the Interaction between Eps-7D and Temperature under Contrasting Photoperiods with Insensitive Ppd-D1 Background
Source: Plants (Basel). 2021 Mar 13;10(3):547. doi: 10.3390/plants10030547 (PMC7999118; doi:10.3390/plants10030547)
Supplement: Supplementary file 1 [file plants-10-00547-s001.pdf]

Wheat developmental traits as affected by the interaction between *Eps-7D* and temperature under contrasting photoperiods with insensitive *Ppd-D1* background

PA Basavaraddi, R Savin, S Bencivenga, S Griffiths, GA Slafer

Supplementary figures

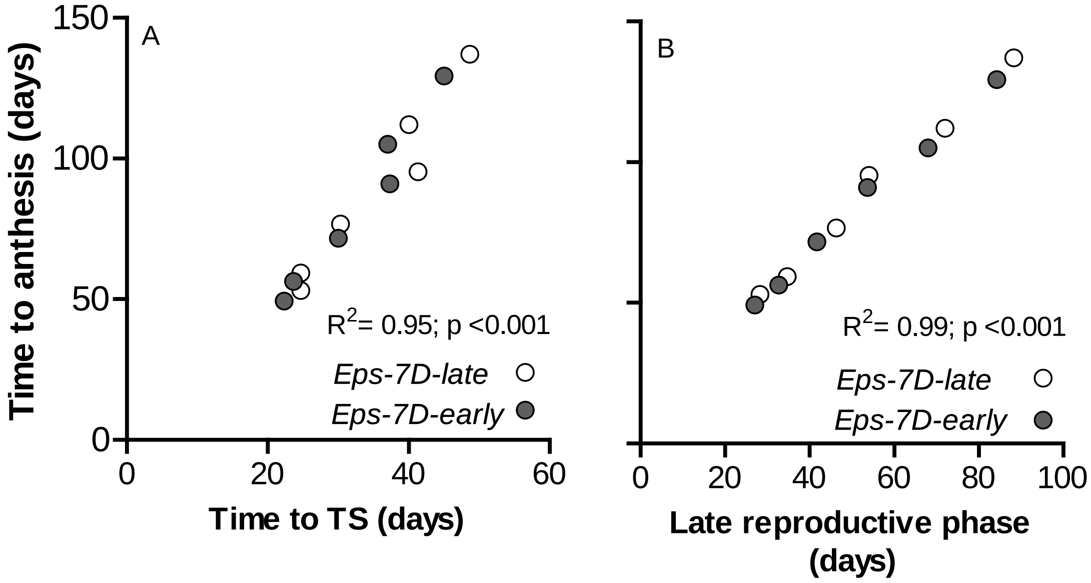

Supplementary Figure S1. Relationships between time from seedling emergence to anthesis and its component phases: time from seedling emergence to terminal spikelet (TS, A) and time from then to anthesis, i.e. the late reproductive phase (B) for the both the NILs carrying either *Eps-7D-late* or *early* allele under three temperatures and two photoperiod regimes.

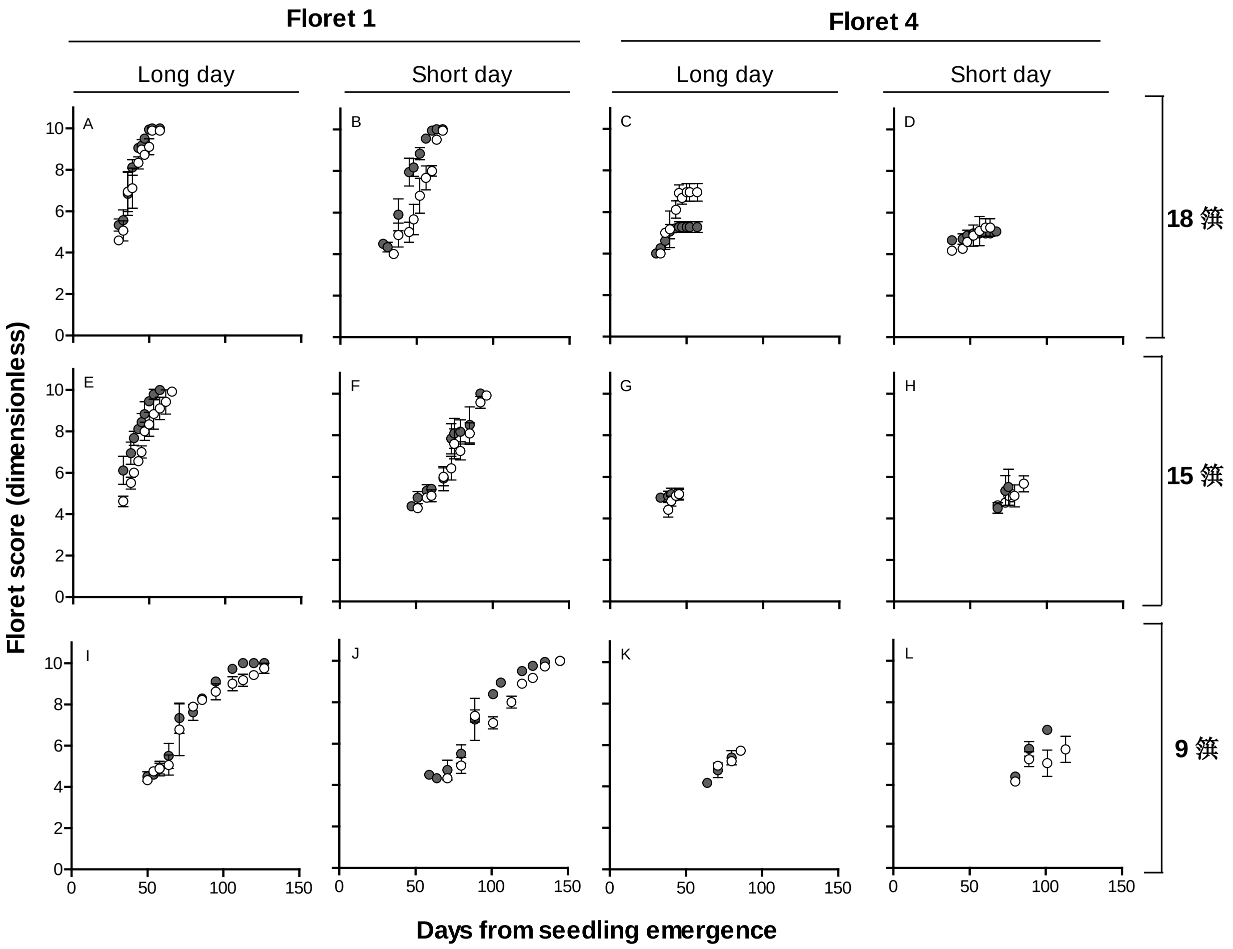

Supplementary Figure S2. Relationship between floret development (floret score, a scale proposed by Waddington et al. [43]) and days from seedling emergence for *Eps-7D*-late (open circles) and *early* (closed circles) for floret 1 (A,B,E,F,I,J) and floret 4 (C,D,G,H,K,L) under long and shot day at 18 (A-D), 15 (E-H) and 9 ºC (I-L). The error bars are standard error of means of floret scores from apical, central and basal spikelets.
